# Solid-State Highly Efficient DR Mono and Poly-dicyano-phenylenevinylene Fluorophores

**DOI:** 10.3390/molecules23071505

**Published:** 2018-06-21

**Authors:** Barbara Panunzi, Rosita Diana, Simona Concilio, Lucia Sessa, Rafi Shikler, Shiran Nabha, Angela Tuzi, Ugo Caruso, Stefano Piotto

**Affiliations:** 1 Department of Agriculture, University of Napoli Federico II, 80055 Portici NA, Italy; barbara.panunzi@unina.it; 2 Department of Chemical Sciences, University of Napoli Federico II, 80126 Napoli, Italy; angela.tuzi@unina.it (A.T.); ugo.caruso@unina.it (U.C.); 3 Department of Industrial Engineering, University of Salerno, 84084 Fisciano SA, Italy; 4 Department of Pharmacy, University of Salerno, 84084 Fisciano SA, Italy; lucsessa@unisa.it (L.S.); piotto@unisa.it (S.P.); 5 Department of Electrical and Computer Engineering, Ben-Gurion University of the Negev, POB 653, Beer-Sheva 84105, Israel; rafi.shikler@googlemail.com (R.S.); nabha@post.bgu.ac.il (S.N.)

**Keywords:** DR, OLED/PLED, PPV, fluorophores, AIE

## Abstract

An efficient deep red (DR)-emitting organic solid based on a dicyano-phenylenevinylene derivative was reported. The structural and spectroscopic properties of the solid have been described in terms of crystallographic data and time-dependent DFT analysis. A noteworthy fluorescence quantum yield of 53% was observed for the brightest emitter cast into solid films. This result can be explained in terms of the aggregation-induced emission (AIE) effect.

## 1. Introduction

Deep red/near-infrared (DR/NIR) solid-state emitters have gained much attention as dopants in fabrication of OLED and PLED devices, in optical probes for bioimaging, and in biomedical applications [1,2,3]. In white PLEDs (WPLEDs), white emission has provided the use of polymer–polymer blends or polymer–small-molecule blends as emissive layers, and the role of the red emitting component is relevant and sensitive [4]. Compared with green and blue emitters [5,6,7,8,9,10,11,12,13], the development of red-light emitting materials is far behind in terms of both purity and efficiency, but is highly required for RGB (red-green-blue)-based devices. In particular, for fluorescence bioimaging and assay applications, the optimal materials are DR/NIR photo emitters with high photoluminescence quantum efficiency, large Stoke’s shifts (for eliminating self-absorption), good chemical and thermal stability, good solubility and processability [14].

So far, the most reported DR/NIR organic emitters consist of a planar polycyclic skeleton having an extended π-system or a strong donor–acceptor framework featuring a large dipole moment in the excited state [15]. NIR emitters are often designed utilizing intramolecular charge-transfer (ICT) to obtain red-shifted emission. Nevertheless, these materials usually tend to aggregate in highly concentrated solutions and in solid states, and this could result in non-uniform thin films and in concentration quenching (aggregation-caused quenching, ACQ effect), due to the strong dipole–dipole interactions. Most dyes are highly emissive in dilute solution but become weakly luminescent or even non-emissive in the solid phase due to the strong dipole–dipole interactions that cause the formation of detrimental excimers and exciplexes. Recently, new types of luminogens showing the aggregation-induced emission (AIE) effect have received much research attention because of their unique optical properties and wide application [16,17,18,19]. The AIE molecules display weak emission when dissolved in solutions, but strong emission in aggregate state, showing the opposite effect of ACQ. Among these molecules, the red emitters are very few, although they are the most promising, for example, in the field of bioimaging, and OLEDs [17,19].

Phenylenevinylene (PV) derivatives are among of the most widely investigated DR/NIR fluorogens. Many oligo or polyphenylenevinylenes (PPVs) have been reported in the literature as emitting layers in OLEDs and PLEDs [20,21,22,23,24,25]. Monomeric and polymeric systems based on PV scaffolds are simple and cheap to realize, processable and easily chemically tuned to give colors in the whole range of the visible spectrum. The introduction of the electron-withdrawing cyano groups on the vinylene moiety leads to an increase in the energy of the occupied π and unoccupied π* states [21], reducing the barrier for electron injection into the conjugated system and improving the electron-transporting properties. This introduction also causes a red shift of the emission maximum compared to less substituted phenylenevinylene systems. Widely reported fully conjugated polymers based on cyano-modified PPV structures feature both good electron transport and efficient emission at high wavelengths in the visible spectrum. In many cases, they represent efficient DR probes with a large Stoke’s shift. The nitrile group provides high electron affinity [26] and exerts a large influence on the photoluminescent (PL) and electroluminescent (EL) properties of the final material [27,28]. Moreover, it can induce steric hindrance, resulting in a twisted conformation, which enables the fluorophores to be protected from the ACQ effect [29,30,31]. Our approach consists in linking well-defined CN-PV emitting units to non-emitting blocks, determining an interruption of the conjugation path. In this way, we can improve the polymer processability as well as prevent the aggregation-caused quenching effect.

In a previous work, we presented a series of blue fluorophores and the related polyamides with the PV units co-polymerized with non-photoluminescent co-monomers [25]. In this article, we describe the facile synthesis of a new PV-based donor-acceptor system (D-A-D-A-D). This probe is symmetrically functionalized with electron-donating terminal amino groups and electron-withdrawing cyano groups on the vinylene moiety. The same diamino precursor was employed to obtain both the final low molecular weight fluorophore and to realize the polyamide. In the latter case, two different dichlorides were simultaneously employed with the aim of obtaining an amorphous ter-copolymer, optimizing both solubility and glass transition temperature. An accurate examination of PL properties in solution and in the solid state has been carried out. Both the fluorophore and the polymer act as DR emitters in the solid state, also showing a large Stoke’s shift and relevant solvatochromic effects. Remarkably, the AIE effect is dominant for the CN-PV-NHMe fluorophore, with a medium quantum efficiency in solution (30%) and a remarkable PLQY (50%) in the solid state. These results are discussed by a correlation of the X-Ray results with a computational study.

## 2. Results and Discussion

### 2.1. General Route for the Synthesis of Fluorophores

**Scheme 1:**
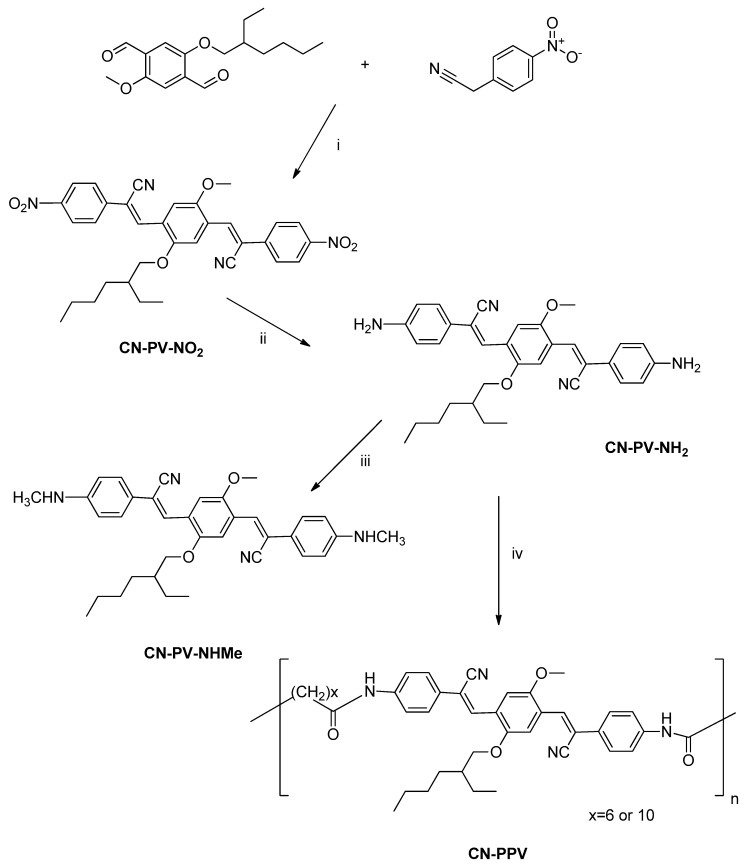
Synthetic route to the target compounds CN-PV-NHMe and CN-PPV. (i) Piperidine, 40 °C, Py, 2 h; (ii) Na_2_S·9H_2_O, DMF/H_2_O, 150 °C, 15 min; (iii) tBOC, dry THF, RT, 6 h; (iv) dodecandioic dichloride, suberic dichloride, THF/dry pyridine, reflux for 2 h.

Mono and polymeric fluorophores were obtained as summarized in Scheme 1. The reduction of CN-PV-NO_2_ led to the monomer CN-PV-NH_2_. The diamino derivative was employed to prepare both the polymer CN-PPV and the compound CN-PV-NHMe, with the same D-A-D-A-D skeleton.

The identification and the evaluation of the purity degree were carried out by mass spectrometry and ^1^H NMR. Phase behavior was examined by optical observation and DSC and TGA analysis. All materials are thermally stable up to 300 °C under nitrogen flow. The ter-polymer CN-PPV was synthesized by polycondensation reaction between the chromophore CN-PV-NH_2_ and a mixture of two different acyl dichlorides (dodecanedioyl and suberoyl). This was designed to guarantee a random and hence more soluble amorphous material, in which chromophores are diluted in a processable polymeric system. Based on T_g_ and η_inh_ values, the polymer can be considered an oligomer, although no evidence of COOH terminal groups was found in the ^1^H NMR spectrum. On the other hand, the low degree of polymerization guarantees good solubility in many common solvents, and therefore easy processability.

### 2.2. Photophysical Properties

**Figure 1:**
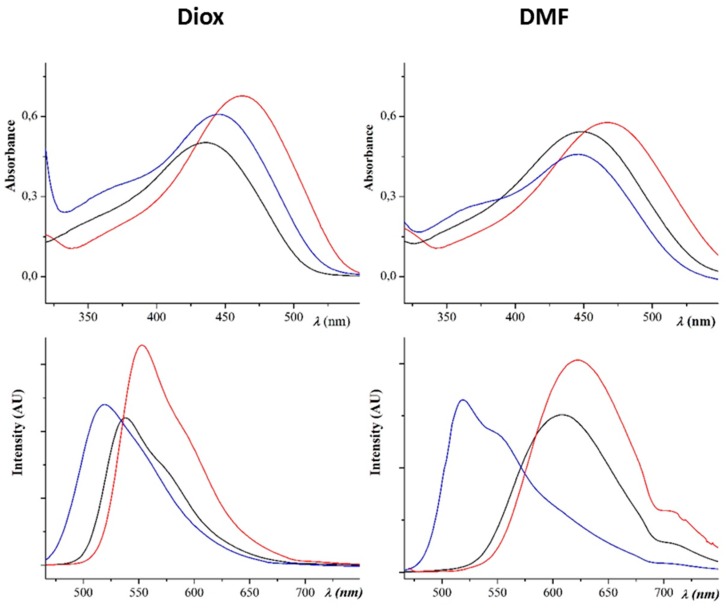
Absorption (**top**) and emission (**bottom**) spectra of CN-PV-NH_2_ (black line), CN-PV-NHMe (red line) and CN-PPV (blue line), in dioxane and DMF solution.

Photophysical measurements were performed both in solution and in the solid state, including PLQY measurements. A strong solvatocromic effect was detected for both of the low molecular weight compounds CN-PV-NH_2_ and CN-PV-NHMe (see Table 1, columns 2 and 3, and Figure 1).

**Figure 2:**
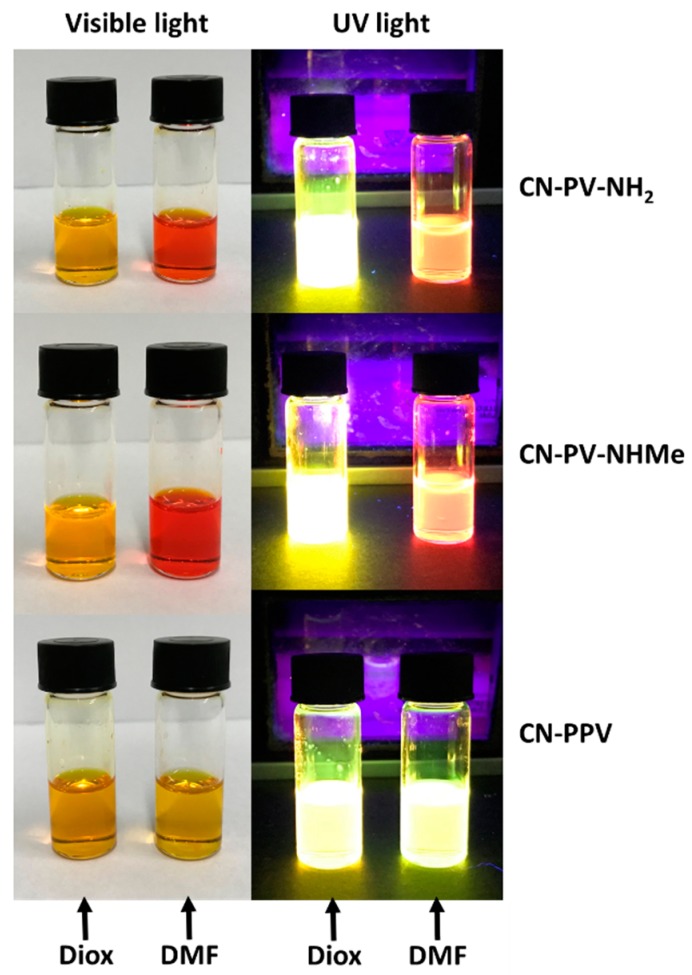
Precursor CN-PV-NH_2_, fluorophore CN-PV-NHMe and polymer CN-PPV solution (10% *w*/*w*) in dioxane (**left**) and DMF (**right**) in natural light (**left column**) and under 375 nm UV light (**right column**).

In solution, the absorption was recorded in the range from 436 to 470 nm and the emission from 518 to 622 nm with a red-shift effect depending on solvent polarity. In dioxane, the compounds appear as yellow-green emitters. In more polar solvents, such as DMF, we observed a red shift of the emission peak. This is consistent with the existence of a polar excited state, which is stabilized in a polar environment and has an increased probability of decaying to the ground state by non-radiative paths (Figure 1). Furthermore, quantum efficiency in DMF decreases significantly. In the case of CN-PPV polymer, we do not observe the same solvatochromic effect: the polymer exhibits a strong yellow-green emission both in dioxane and in DMF, with CIE 1931 coordinates of (0.36, 0.57) and a measured PLQY of 26% in dioxane or in DMF solution (see Figure 2).

**Figure 3:**
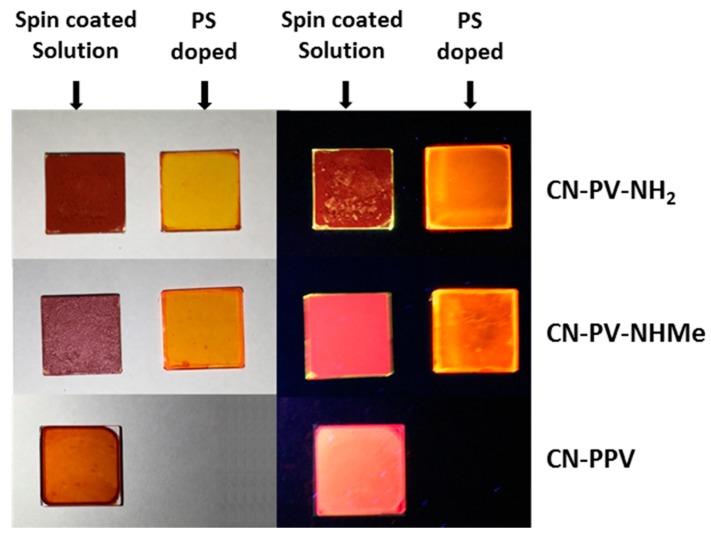
Fluorophore thin films (**left**) and in 10% PS-doped thin film (**right**) in natural (**left column**) and under 375 nm UV light (**right column**).

In solution, PL quantum yields were measured by relative methods, using as standard quinine sulfate QS (UQS ¼ 0.546 in H_2_SO_4_ 1 N, when excited at 365 nm according to Melhuish [32]). The values appear to be independent of the solvent, and are very similar. All compounds show deep red photoluminescence with a pronounced Stoke’s shift both in solution and in the solid state. Remarkably, in the solid state, Stoke’s shifts range from 140 to 160 nm. In order to test the effect of a dilution in the solid state, we prepared two types of solid films: by spin-coating a solution of each compound and by microdispersion of the shattered crystals in a polystyrene matrix at 10% by weight (PS-doped film). In some cases, the use of a microdispersion of the crystalline fluorophores in PS represented the right way to prevent the ACQ effect in the solid state [14,24]. In Figure 3, the solid samples on quartz slides are shown.

All solid compounds are red, both under natural light and UV lamp. CN-PV-NH_2_ micro-dispersed in polystyrene (Figure 3, top) showed enhancement of PL emission respect to the pure thin film, reaching the same value when measured in dioxane-diluted solution (see also Figure 2). This is evidence of an ACQ effect in the pure solid film for this compound. The ACQ effect is lowered in the PS-doped film, thanks to the dilution of the fluorophores in the PS matrix. CN-PV-NHMe displays a deep red emission, with a remarkable 53% PLQY in thin film. The same compound dispersed in polystyrene emits at 598 nm with a comparable 50% PLQY, whereas in solution, it shows only a faint emission. The stronger emission observed in the solid state could be ascribable to the presence of cyano substituents on the conjugated system, whose effect is to restrict molecular motions and to stabilize a twisted conformation in the solid. This permits the fluorophore to emit more efficiently in the aggregated state. In fact, the twisted geometry, hindering intermolecular close stacking and intense π-π interaction, may lead to intense aggregation-induced emission effect (AIE) [16]. In order to verify this hypothesis, we carried out a crystallographic study on CN-PV-NHMe, which shows a higher PLQY in the solid and was easily obtained as single crystals.

In the solid phase, the polymer is also a DR emitter (see Figure 3), but with a PLQY value similar to those of the compounds in solution (Table 1). CN-PPV can be considered, in fact, a diluted system of the fluorophore core. The PLQY value measured for CN-PPV thin film is lower than the value of CN-PV-NHMe and close to the value of CN-PV-NH_2_ dispersed in polystyrene; this is not surprising, because in the polymer chain the fluorophore replaces its strong electron donating amino groups with amide moieties, which possess a lower electron-donating ability. This result, however, is not easily rationalizable, because dipole–dipole interactions in the polymeric amorphous sample are conceivably very different from the same interactions acting in the crystalline samples.

### 2.3. X-ray Crystallography

**Figure 4:**
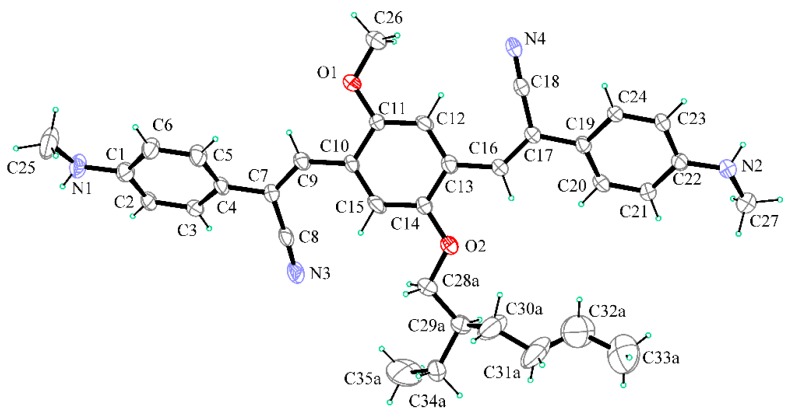
Ortep view of CN-PV-NHMe with thermal ellipsoids drawn at 30% probability level. Only the major part of the disordered aliphatic group is shown for clarity.

The molecular structure of CN-PV-NHMe was determined by single crystal X-ray analysis. Compound crystallizes in the monoclinic C2/c space group, with one molecule contained in the asymmetric unit. All bond lengths and angles fall within the normal range. An ORTEP view of the molecular structure is reported in Figure 4. Details of crystallographic data and refinement parameters are reported in Table S1 of the Supplementary Materials.

**Figure 5:**
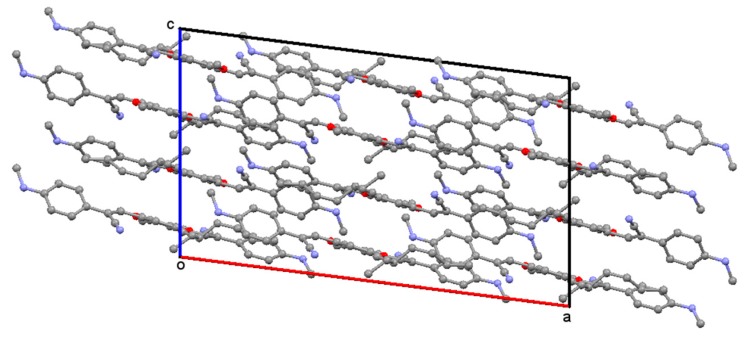
Crystal packing of CN-PV-NHMe viewed along **b** axis. Ball-and-stick style, H atoms not drawn for clarity.

The molecule is characterized by three phenyl rings mutually twisted each to the other. The mean planes of outlying C1/C6 and C19/C24 phenyl rings are tilted 49.01(18)° and 31.47(16)° with respect to the central C10/C15 mean plane ring. An overall elongated, almost flat, shape of the molecule is observed, with aminic and alifatic groups being quite in-plane. As expected, the planar geometry at the aminic N atoms shows a degree of conjugation with the bound phenyl group. The crystal packing is stabilized by face-to-edge aromatic interactions (Figure 5). No π-π aromatic stacking is found, due to the twisting of phenyl rings in adjacent molecules (Figure S1, Supplementary Material).

In the crystal packing, the aminic hydrogen atoms are involved in intermolecular weak interactions with the -CN groups of adjacent molecules, forming linear ribbons of molecules in the (1 − 1 0) and (1 1 0) directions (N1-H···N4^i^: 0.97(6) Å, 2.34(6) Å, 3.237(7) Å, 153(5)°, i = x + 1/2, y − 1/2, z; N2-H···N3^ii^ 0.84(5) Å, 2.51(5) Å, 3.323(7) Å, 166(5)°, ii = x − 1/2, y + 1/2, z) (See Figure S2, Supplementary Material).

### 2.4. Computational Studies

**Figure 6:**
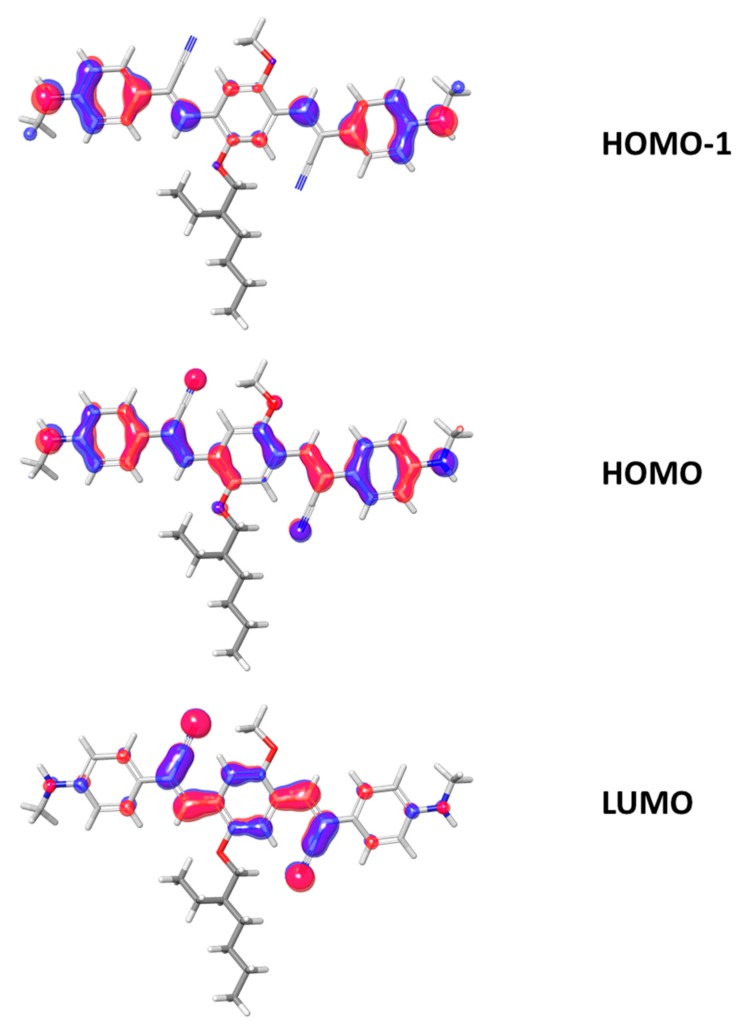
HOMO-1, HOMO and LUMO representation for CN-PV-NHMe.

The excitation energies were obtained at the density functional level by using the time-dependent perturbation theory approach (TDDFT) with the adiabatic local density approximation, which have high reliability in obtaining accurate predictions for excitation energies and oscillator strengths. Calculations on CN-PV-NHMe in DMF showed the localization of HOMO and LUMO orbitals (see Figure 6).

HOMO is delocalized over the entire conjugated backbone, with only a moderate contribution of the cyano groups. Contrarily, the LUMO is mainly focused on the central part of the molecule and on the electron-withdrawing cyano groups. The main transition, at 487 nm, is the HOMO → LUMO. The second strongest absorption at 394 nm is due to the transition HOMO–1 → LUMO. The predicted absorption maximum is in good agreement with the experimental values (see Table 1 and Table 2), the two main absorptions correspond to the HOMO → LUMO and HOMO-1→LUMO transitions.

CN-PV-NHMe exhibits hole and electron reorganization energies (HRE and ERE respectively) that are typical for these compounds. DFT calculations make is possible to investigate the first oxidation potential to explain the electron-donating properties of the compound. Withdrawal of the electron from the compound in a redox reaction is performed from its HOMO. The observed trend in oxidation potential for the three compounds indicates destabilization of the HOMO. The intense DR emission of CN-PV-NHMe arises from the D-A-D-A-D motif with strong donor and acceptor functionalities and, at the same time, largely delocalized frontier MOs, giving rise to the high oscillator strength of the emitting state. As reported for similar compounds [14], in our systems, only a minor intramolecular charge transfer character is observed, and the oscillator strength is thus large for the ground to the first excited state transition (f = 2.27) [31]. The large radiative decay and the strong fluorescence, with a quantum yield of about 50% in the solid, could also be attributed to strongly twisted equilibrium structures, as confirmed by the X-ray analysis (Figure 4 and Figure 5).

## 3. Materials and Methods

All starting products were commercially available. The chlorides were distilled before use. 2-(2-Ethylhexyloxy)-5-methoxyterephthalaldehyde was obtained as described in [25]. Optical observations were performed by using a Zeiss Axioscop polarizing microscope equipped with a FP90 Mettler heating stage. Phase transition temperatures and enthalpies were measured using a DSC scanning calorimeter Perkin Elmer Pyris 1 at a scanning rate of 10 °C/min, under nitrogen flow. The decomposition temperature (T_d_) is assumed as the temperature where is recorded the 5 wt. % weight loss. T_d_ were determined by thermogravimetric analysis under nitrogen flow, using a Perkin Elmer TGA 4000. ^1^H NMR spectra were recorded with Bruker Avance II 400 MHz apparatus. Mass spectrometry measurements were performed using a Q-TOF premier instrument (Waters, Milford, MA, USA) equipped by an electrospray ion source and a hybrid quadrupole-time of flight analyzer. Mass spectra were acquired in positive ion mode, in 50% CH_3_CN solution, over the 400–800 *m*/*z* range. Instrument mass calibration was achieved by a separate injection of 1 mM NaI in 50% CH_3_CN. Data were processed by using MassLynx software (Waters). UV-Visible and fluorescence spectra were recorded with JASCO spectrometers. Jasco F-530 (scan rate 200 nm min^−1^) and on a spectrofluorimeter Jasco FP-750 (excitation wavelengths set at the absorption maxima of the samples, scan rate 125 nm min^−1^). For the polymer CN-PPV, Inherent viscosity (η_inh_) at 25.0 °C in N-methyl pyrrolidinone (NMP) was measured with an Ubbelohde viscometer. The molecular mass and its distribution was obtained with a Lab Flow 2000 GPC/HPLC apparatus with polystyrene samples as references and tetrahydrofuran (THF) as the eluent.

Thin films of the amino precursor, the fluorophore and the polymer were obtained by the spin-coating technique of a micro-dispersion of shattered crystals (10% by weight) and low weight commercially available polystyrene (PS) in acetone/dichloromethane (1:2) by a SCS P6700 spin-coater operating at 800 RPM. Thin films of the sample without polystyrene were obtained by casting from a chloroform solution (about 30% by weight).

### 3.1. Preparation of CN-PV-NO_2_

2-(2-Ethylhexyloxy)-5-methoxyterephthalaldehyde (7.60 g, 0.0260 mmol) [25] and piperidine (2 mL) were dissolved in pyridine (20 mL). The solution was warmed at 40 °C and then 2-(4-nitrophenyl)acetonitrile (16.2 g, 0.102 mol) was added in portions over 1 h. After this time, the solution color turned to red and the formation of a solid occurred. After one more hour, the reaction was stopped and the suspension was poured in 40 mL of ethanol at room temperature. The bright red solid was then recovered by filtration. The compound was recrystallized from ethanol in needle-shaped, dark red crystals. Yield: 55%; mp: 232 °C; T_d_ = 352 °C. ^1^H NMR (400 MHz, DMSO-*d*_6_, 25 °C, ppm): 0.84 (m, 6H), 1.31 (m, 8H), 1.75 (m, 1H), 3.91 (s, 3H), 4.01 (d, *J* = 4.8 Hz, 2H), 7.81 (d, *J* = 3.2 Hz, 4H), 8.00 (t, *J* = 8.4 Hz, 2H), 8.26 (d, *J* = 4.0 Hz, 4H), 8.32 (m, 2H). ^13^C NMR (400 MHz, DMSO-*d*_6_, 25 °C, ppm): 12.5 (1C), 14.6 (1C), 22.8 (1C), 24.6 (1C), 30.7 (1C), 31.4 (1C) 38.8 (1C), 57.5 (1C), 70.7 (1C), 114.7 (2C), 116.9 (2C), 119.7 (4C), 123.8 (2C), 127.7 (4C), 139.8 (4C), 149.5 (2C), 152.1 (2C), 152.7 (2C). HRMS(ESI): *m*/*z* calcd for C_33_H_32_N_4_O_6_ + H^+^: 581.23; found 581.12 [M + H]^+^

### 3.2. Preparation of CN-PV-NH_2_

To CN-PV-NO_2_ (1.00 g, 0.700 mmol) dissolved in DMF (20.0 mL) 5.60 mL of a 1.20 M Na_2_S·9H_2_O aqueous solution was added and the color turned from red to black. After 15 min, water (1.00 mL) was added and the system kept at 150 °C for 15 min. The mixture was slowly cooled to room temperature, leading to the formation of a red crystalline solid. The compound was recovered by filtration and washed with slightly acid water (acetic acid, pH about 5) and then with water. The red crystalline product was crystallized again from chloroform/ethanol. Yield: 90%; mp: 190 °C; T_d_ = 337 °C. ^1^H NMR (400 MHz, DMSO-*d*_6_, 25 °C, ppm): 0.96 (m, 6H), 1.48 (m, 8H),1.90 (m, 1H), 3.96 (s, 3H), 4.06 (m, *J* = 4.0 Hz, 2H), 5.79 (s, 4H), 6.80 (d, *J* = 8.2Hz, 4H), 7.49 (m, 4H), 7.85 (m 4H). ^13^C NMR (400 MHz, DMSO-*d*_6_, 25 °C, ppm): 13.9 (1C), 14.1 (1C), 23.3 (1C), 24.1 (1C), 30.1 (1C), 30.7 (1C) 40.1 (1C), 58.3 (1C), 69.4 (1C), 113.1 (1C), 114.5 (1C), 115.5 (2C), 119.3 (4C), 121.2 (2C), 126.7 (2C), 129.1 (2C), 132.5 (4C), 143.2 (2C), 151.3 (2C), 156.8 (2C). HRMS(ESI): *m*/*z* calcd for C_33_H_36_N_4_O_2_ + H^+^: 521.28; found 521.30 [M + H]^+^

### 3.3. Preparation of CN-PV-NHMe

To 1.35 g (5.37 mmol) of CN-PV-NH_2_, suspended in 10 mL of dry THF, 2.79 g (16.0 mmol) of terzbutyl carbonate (tBOC) was added under stirring at room temperature. After 6 hours, the solvent was removed in vacuo and the crude product washed with hot hexane three times. The compound was employed in the following step without any further purification.

To CN-PV-NHtBOC dissolved in 200 mL of boiling acetone, 8.10 g (145 mmol) of KOH powder was slowly added, followed by a first aliquot of 7.70 mL of ICH_3_. Then a second aliquot of ICH_3_ (7.70 mL) was added dropwise after 15 min. The reaction was kept to the boil under stirring for 15 min. The mixture was cooled at room temperature, filtered and the solution concentered in vacuo to 50 mL and 100 mL of heptane added, with the precipitation of a red-orange solid ensuing. The product was recovered by filtration and crystallized from acetone/heptane. Yield: 70%; mp: 160 °C; T_d_ = 367 °C. ^1^H NMR (400 MHz, DMSO-*d*_6_, 25 °C, ppm): 0.90 (m, 6H), 1.46 (m, 8H), 1.77 (m, 1H), 2.90 (s, 6H), 3.94 (s, 3H), 4.01 (m, 2H), 6.28 (m, 4H), 6.68 (m, 4H), 7.55 (m, 2H), 7.85 (m, 4H). ^13^C NMR (400 MHz, DMSO-*d*_6_, 25 °C, ppm): 13.6 (1C), 13.9 (1C), 22.8 (1C), 23.1 (1C), 30.1 (1C), 30.9 (1C) 32.0 (2C), 40.0 (1C), 56.5 (1C), 70.3 (1C), 111.4 (1C), 112.9 (4C), 113.7 (1C), 118.5 (2C), 119.8 (2C), 121.7 (2C), 128.7 (4C), 129.8 (2C), 146.5 (2C), 153.2 (2C), 157.5 (2C). HRMS(ESI): *m*/*z* calcd for C_35_H_40_N_4_O_2_ + H^+^: 549.32; found 549.30 [M + H]^+^

### 3.4. Preparation of CN-PPV

To 0.160 g (0.60 mmol) of dodecandioic dichloride and 0.126 g (0.60 mmol) of suberic dichloride dissolved in 5.0 mL of THF a solution of CN-PV-NH_2_ (0.607 g, 1.20 mmol) dissolved in 5 mL of dry pyridine was added. The reaction was kept under vigorous stirring at reflux for 2 h. The solution was poured into 150 mL of water and a solid product was recovered by filtration. The red crude product was dissolved in hot DMF (50 mL) and precipitated again in 400 mL of water, recovered by filtration and dried at 120 °C for 4 h. Yield: 80%; T_g_ = 112 °C; T_d_ = 385 °C. η_inh_ (DMF, 25 °C) = 0.50 dL/g. M_n_ = 5800 Da, polydispersity indexes (M_w_/M_n_) = 2.2. ^1^H NMR (400 MHz, DMSO-*d*_6_, 25 °C, ppm): 0.85–0.95 (m, 6H), 1.31–1.61 (m, 20H), 2.30–2.46 (m, 5H), 3.96 (m, 5H), 5.70 (m, 2H), 6.66 (m, 2H), 7.76 (m, 8H), 10.17 (s, 2H). ^13^C NMR (400 MHz, DMSO-*d*_6_, 25 °C, ppm): 12.5 (1C), 13.4 (1C), 23.1 (1C), 23.5 (1C), 26.1 (2C), 29.8 (2C), 30.3 (4C), 32.4 (2C), 35.2 (2C), 40.7 (1C), 55.6 (1C), 71.4 (1C), 118.8 (4C), 119.7 (2C), 120.4 (2C), 122.5 (2C), 127.9 (4C), 147.2 (2C), 149.4 (2C), 156.3 (2C), 173.5 (2C), 182.7 (2C).

### 3.5. PLQY Measurements

Photoluminescence efficiency measurements were conducted with a setup similar to the one suggested by de Mello et al. [33]. This setup takes into account not only the exciting laser and direct photoluminescence spectrum but also the photoluminescence that originates due to the scattering effect of the integrating sphere that is part of the setup. The setup consists of a 376 nm laser whose emission does not overlap with the emitted photoluminescence spectrum, an integrating sphere (Stellarnet Inc, Tampa, FL, USA) and a photospectrometer (BLACK Comet Stellarnet Inc, Tampa, FL, USA). The emission was measured at five different points on the sample.

### 3.6. X-ray Crystallography

Single crystals of CN-PV-NHMe were obtained by slow evaporation of acetone/heptane solution at ambient temperature. It was not possible to obtain very good quality crystals. One selected crystal was mounted in flowing N_2_ at 173 K on a Bruker-NoniusKappaCCD diffractometer equipped with Oxford Cryostream apparatus (graphite monochromated MoK_α_ radiation, λ = 0.71073 Å, CCD rotation images, thick slices, φ and ω scans to fill asymmetric unit). Semi-empirical absorption corrections (SADABS [34]) were applied. The structure was solved by direct methods (SIR97 program [35]) and anisotropically refined by the full matrix least-squares method on F^2^ against all independent measured reflections using SHELXL-2016 [36] and WinGX software [37]. The H atoms at aminic groups were located in difference Fourier maps and freely refined with U_iso_(H) equal to 1.2U_eq_ of the carrier atom. All the other hydrogen atoms were introduced in calculated positions and refined according to the riding model with C–H distances in the range 0.95–1.00 Å and with U_iso_(H) equal to 1.2U_eq_ or 1.5U_eq_(C_methyl_) of the carrier atom. Difference Fourier maps showed two disordered positions of the branched aliphatic group that refined with occupancy factors 0.596 and 0.404. Some restraints were introduced in the last stage of refinement to model the disorder using DFIX, SAME and SIMU commands of SHELXL program. Crystal data and structure refinement details are reported in Table S1 (Supplementary Materials). The figures were generated using ORTEP-3 [38] and Mercury CSD 3.9 [39] programs.

Crystallographic data were deposited at Cambridge Crystallographic Data Centre with assigned number CCDC 1843139. These data can be obtained free of charge from www.ccdc.cam.ac.uk/structures.

### 3.7. Computational Studies

All quantum calculations were carried out using the Jaguar program [40], as implemented in the Schrödinger Material Science Suite [41]. Geometry optimizations were performed with the B3LYP functional and the 6-31G** basis set in which the transition metals are represented using the Los Alamos LACVP** basis that includes relativistic effective core potentials. The energies of the optimized structures are re-evaluated by additional single-point calculations on each optimized geometry using Dunning’s correlation-consistent triple-ζ basis set42 cc-pVTZ(-f), which includes a double set of polarization functions. Vibrational frequency calculation results based on analytical second derivatives at the B3LYP/6-31G**(LACVP**) level of theory were used to confirm proper convergence to local minima and to derive the zero-point-energy (ZPE) and entropy corrections at room temperature where unscaled frequencies were used.

## 4. Conclusions

A new DR dicyano-phenylenevinylene emitter and the ter-polymer containing the same fluorophore unit have been synthesized. A detailed study of PL properties in solution and in the solid-state compounds has been carried out. A bright red emission for the fluorophore was recorded with 50% PLQY in the solid state. Interestingly, no ACQ effect was detected on the fluorophore. A detailed X-ray investigation correlated with TD-DFT analysis was presented in order to explore this behavior. In the solid state of CN-PV-NHMe (film or doped PS film), owing to the restriction of intramolecular motions induced by the cyano substituents, the radiative channel was activated, causing intense aggregation-induced emission (AIE). Regarding the polymer, although a noteworthy quantum efficiency was not observed, this was a way of exploiting the advantages to covalently bond the fluorophores into an easily processable material retaining medium DR photoluminescence.

## Figures and Tables

**Table 1:** Optical data of synthesized compounds.

Compound	λ_abs-sol_ (nm) ^a^	λ_em-sol_ (nm) ^b^	λ_abs-film_ (nm) ^c^	λ_em-film_ (nm) ^d^	PLQY% Film ^e^	PLQY% Solution ^f^
CN-PV-NH_2_	436 ^i^; 449 ^ii^	536 ^i^; 609 ^ii^	476 ^j^; 462 ^jj^	530 ^j^; 559 ^jj^	5 ± 1 ^j^; 27 ± 5 ^jj^	26 ± 3
CN-PV-NHMe	464 ^i^; 470 ^ii^	554 ^i^; 622 ^ii^	495 ^j^; 474 ^jj^	635 ^j^; 598 ^jj^	53 ± 5 ^j^; 50 ± 8 ^jj^	29 ± 3
CN-PPV	441 ^i^; 445 ^ii^	518 ^i,ii^	458 ^j^	613 ^j^	18 ± 3 ^j^	26 ± 3

^a^ Wavelength of UV-Visible absorbance maxima in ^(i)^ dioxane ^(ii)^ DMF solution. ^b^ Wavelength of emission maxima in ^(i)^ dioxane ^(ii)^ DMF solution. ^c^ Wavelength of UV-Visible absorbance maxima on thin film obtained ^(j)^ by slow evaporation of the sample or ^(jj)^ by dispersing the sample in polystyrene at 10% by weight. ^d^ Wavelength of emission maxima on thin film obtained ^(j)^ by slow evaporation of the sample or ^(jj)^ by dispersing the sample in polystyrene at 10% by weight. ^e^ PL quantum yield on thin film obtained ^(j)^ by slow evaporation of the sample or ^(jj)^ by crystalline micro-dispersion in polystyrene, 10% by weight. ^f^ PL quantum yield in dioxane.

**Table 2:** Calculated electro-optical parameters for CN-PV-NHMe.

**Oxidation Potential (eV)**	**Reduction Potential (eV)**	**Hole Reorganization Energy (eV)**	**Electron Reorganization Energy (eV)**	**Triplet Energy (eV)**	**λ_abs_ Solution (nm)**	**λ_em_ Solution (nm)**	**Triplet Stabilization Energy (eV)**
−0.022	−1.754	0.229	0.457	1.477	487	587	0.313
**Triplet Reorganization Energy (eV)**	**Electron Small Polaron Stabilization Energy (eV)**	**Hole Extraction Potential (eV)**	**Electron Extraction Potential (eV)**	**Hole Small Polaron Stabilization Energy (eV)**	**Dipole (D)**	**Scaled HOMO (eV)**	**Scaled LUMO (eV)**
0.603	0.328	5.429	−1.330	0.116	0.420	−4.508	−2.776

## Supplementary Materials

The following are available online, Table S1: Crystallographic data and structural refinement details of CN-PV-NHMe. Figure S1: Partial packing of CN-PV-NHMe with shortest distances involving aromatic centroids. Figure S2: Molecular ribbon of CN-PV-NHMe propagating in the (1 –1 0) direction. Figure S3: ^1^H NMR spectra of CN-PV-NH_2_, CN-PV-NHMe and CN-PPV.
