# Supplementary material for: Solid-State Highly Efficient DR Mono and Poly-dicyano-phenylenevinylene Fluorophores
Source: Molecules. 2018 Jun 21;23(7):1505. doi: 10.3390/molecules23071505 (PMC6099678; doi:10.3390/molecules23071505)
Supplement: Supplementary file 1 [file molecules-23-01505-s001.pdf]

Solid-state highly efficient DR mono and poly-dicyano-phenylenevinylene fluorophores

Barbara Panunzi [1], Rosita Diana [2,*], Simona Concilio [3,*], Lucia Sessa [4], Rafi Shikler [5], Shiran Nabha [5], Angela Tuzi [2], Ugo Caruso [2] and Stefano Piotto [4]

[1] Department of Agriculture, University of Napoli Federico II, Portici NA, Italy
[2] Department of Chemical Sciences, University of Napoli Federico II, Napoli, Italy
[3] Department of Industrial Engineering, University of Salerno, Fisciano SA, Italy
[4] Department of Pharmacy, University of Salerno, Fisciano SA, Italy
[5] Department of Electrical and Computer Engineering, Ben-Gurion University of the Negev, Israel.

* Correspondence: rosita.diana@libero.it; sconcilio@unisa.it; Tel.: +39-089-964115

Table S1. Crystallographic data and structural refinement details of CN-PV-NHMe.

Empirical formula	$C_{35}H_{40}N_4O_2$
Formula weight	548.71
Temperature (K)	173(2)
Wavelength (Å)	0.71073
Crystal system	Monoclinic
Space group	C 2/c
a (Å)	25.330(7)
b (Å)	16.719(4)
c (Å)	14.804(3)
β (°)	97.269(18)
Volume (Å^3)	6219(3)
Z	8
D_{calc} (Mg/m^3)	1.172
μ (mm^{-1})	0.073
F(000)	2352
Crystal size (mm)	0.40 × 0.10 × 0.07
θ range for data collection (°)	2.720 to 25.013
Limiting indices	$-30 \le h \le 30$, $-19 \le k \le 19$, $-17 \le l \le 16$
Reflections collected / unique	27781 / 5463 [*R*(int) = 0.2470]
Refinement method	Full-matrix least-squares on F^2
Data / restraints / parameters	5463 / 200 / 443
Goodness-of-fit on F^2	1.015
Final *R* indices [$I>2\sigma(I)$]	R_1 = 0.0833, wR_2 = 0.1817
R indices (all data)	R_1 = 0.2390, wR_2 = 0.2521
Largest diff. peak / hole (e·A^{-3})	0.242 / -0.240

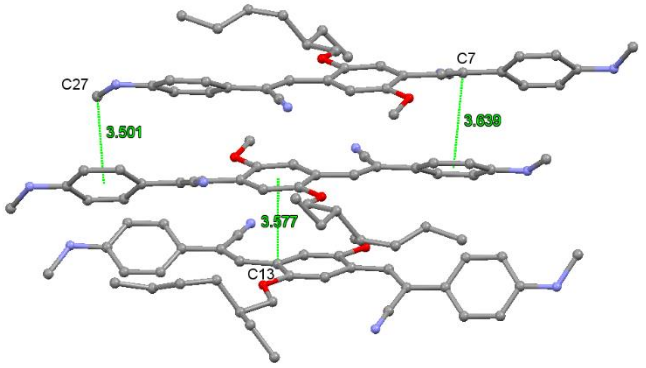

Figure S1. Partial packing of CN-PV-NHMe with shortest distances involving aromatic centroids reported as green dashed lines. Ball-and-stick style, H atoms are not drawn for clarity.

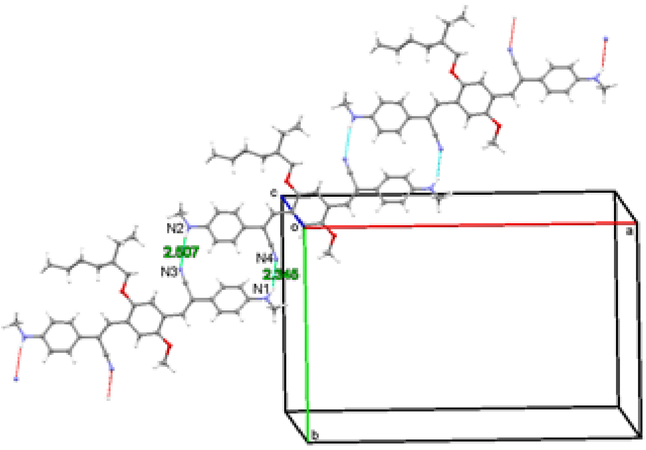

Figure S2. Molecular ribbon of CN-PV-NHMe propagating in the (1 -1 0) direction. Intermolecular NH···N bonds are reported as light blue dahsed lines.

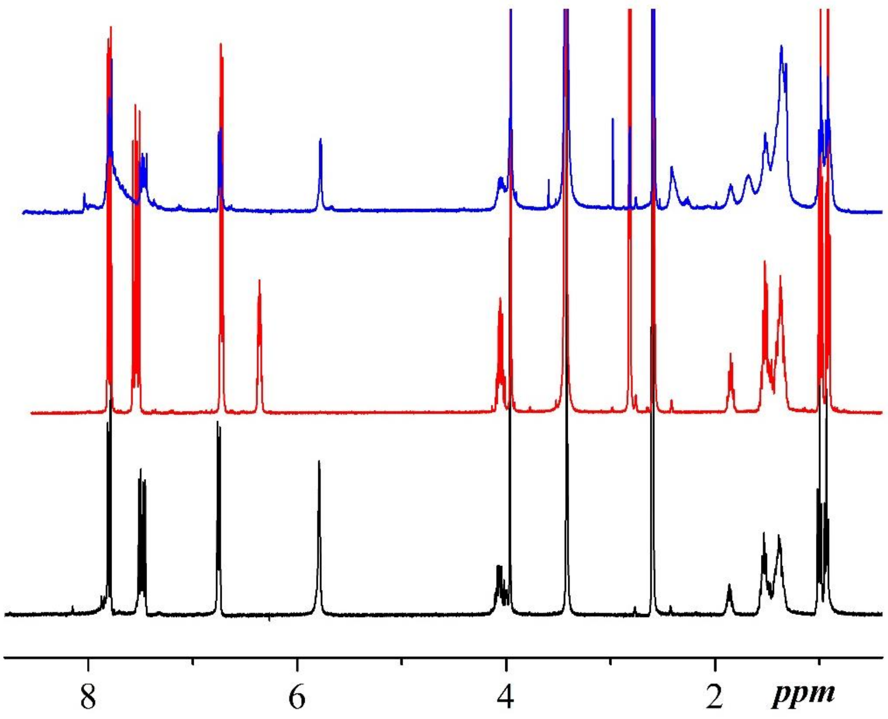

Figure S3. ^1H NMR spectra of CN-PV-NH_2 (black line), CN-PV-NHMe (red line) and CN-PPV (blue line) in DMSO-d_6, 400 MHz, 25°C.
